
JOURNAL INFORMATION
==============================
NLM Title Abbreviation: Biospektrum (Heidelb)
Journal ID: Biospektrum (Heidelb)
NLM Journal ID: 9615685

Biospektrum

ISSN: 0947-0867
EISSN: 1868-6249

ARTICLE INFORMATION
==============================
PMCID: PMC8960702
PMID: 35369113
DOI: 10.1007/s12268-022-1730-9
Article ID: 1730
Article version: 1
Subjects: Wissenschaft · Special: Virologie

Möglicher Einfluss von Viren auf die Ausbreitung von Proteinaggregaten

Heumüller Stefanie-Elisabeth
Vorberg Ina Maja ina.vorberg@dzne.de

grid.424247.3 0000 0004 0438 0426 Deutsches Zentrum für Neurodegenerative Erkrankungen, Venusberg-Campus 1/99, D-53127 Bonn, Deutschland
Electronic publication date: 2022 Mar 28
Print publication date: 2022
Volume: 28
Issue: 2
First page: 162
Last page: 164
Copyright: © Die Autorinnen 2022
License: Open Access: Dieser Artikel wird unter der Creative Commons Namensnennung 4.0 International Lizenz veröffentlicht, welche die Nutzung, Vervielfältigung, Bearbeitung, Verbreitung und Wiedergabe in jeglichem Medium und Format erlaubt, sofern Sie den/die ursprünglichen Autor(en) und die Quelle ordnungsgemäß nennen, einen Link zur Creative Commons Lizenz beifügen und angeben, ob Änderungen vorgenommen wurden. Die in diesem Artikel enthaltenen Bilder und sonstiges Drittmaterial unterliegen ebenfalls der genannten Creative Commons Lizenz, sofern sich aus der Abbildungslegende nichts anderes ergibt. Sofern das betreffende Material nicht unter der genannten Creative Commons Lizenz steht und die betreffende Handlung nicht nach gesetzlichen Vorschriften erlaubt ist, ist für die oben aufgeführten Weiterverwendungen des Materials die Einwilligung des jeweiligen Rechteinhabers einzuholen. Weitere Details zur Lizenz entnehmen Sie bitte der Lizenzinformation auf http://creativecommons.org/licenses/by/4.0/deed.de.
License URL: https://creativecommons.org/licenses/by/4.0/

issue-copyright-statement: © The Author(s), under exclusive licence to Springer-Verlag GmbH Deutschland, ein Teil von Springer Nature 2022

==============================
Neurodegenerative diseases are associated with misfolding of proteins into highly-ordered amyloid fibrils. These protein aggregates can be transmitted to other cells in which they induce aggregation of proteins of the same kind. Mechanisms of intercellular transfer include direct cell contact or transfer of aggregates within extracellular vesicles. Recent research suggests that viral proteins can increase the intercellular spreading of protein aggregation by promoting the required membrane interactions.

■ In unserer alternden Gesellschaft zählen neurodegenerative Erkrankungen wie die Alzheimer-Erkrankung zu den größten medizinischen Herausforderungen der kommenden Jahrzehnte. Die Anzahl an Demenzkranken allein in Deutschland wird auf 1,7 Millionen geschätzt [1]. Anomale Ablagerungen zellulärer Proteine (Proteinaggregate) innerhalb und außerhalb von Zellen des zentralen Nervensystems (ZNS) sind charakteristisch für neurodegenerative Erkrankungen [2]. Intrazelluläre Ablagerungen des fehlgefalteten mikrotubulibindenden Proteins Tau finden sich bei einer heterogenen Gruppe neurodegenerativer Erkrankungen, den Tauopathien, zu denen auch die Alzheimer-Erkrankung gehört. Die genauen Abläufe, welche zur Proteinaggregation führen, sind unbekannt.

## Proteinaggregate können sich von Zelle zu Zelle ausbreiten

Die Proteinaggregate entstehen nach dem Prinzip der keiminduzierten Polymerisierung. Hierbei lagern sich bestimmte krankheitsassoziierte Proteine, wie Tau, spontan zu Oligomeren zusammen. Im Anschluss kommt es zur Ausbildung von Amyloidfibrillen, in denen die Monomere über Betafaltblattstrukturen geordnet miteinander assoziieren. Fibrillen und Bruchstücke induzieren als Aggregationskeime die Fehlfaltung weiterer Proteinmonomere (**Abb. 1**). Interessanterweise scheinen bereits gebildete Proteinaggregate die Eigenschaft zu haben, sich innerhalb des Gehirns von Zelle zu Zelle auszubreiten. So postulierten H. Braak *et al.*, dass sich krankhafte Proteinablagerungen ausgehend von definierten Bereichen des Gehirns im Verlauf der Erkrankung über weitere Hirnareale nach typischen Mustern ausbreiten [3]. Zellkulturmodelle, die in den letzten Jahren entwickelt wurden, haben die Übertragungswege zwischen Zellen genauer beleuchtet. So scheinen sowohl der direkte Zellkontakt als auch die Freisetzung „nackter" Proteinaggregate in den extrazellulären Raum und die Verpackung der Proteinaggregate in extrazelluläre Vesikel (EV) mögliche Wege darzustellen (**Abb. 2A**). Bei EV handelt es sich um Vesikel, die für die interzelluläre Kommunikation freigesetzt werden. Offensichtlich ist, dass in zwei der drei Szenarien Membranen miteinander in Kontakt treten und verschmelzen müssen, um Proteinaggregate in das Cytosol der Empfängerzelle freizusetzen. Die für den Zellkontakt und die Aufnahme von EV notwendigen Liganden- und Rezeptorpaare sind jedoch meist unbekannt.

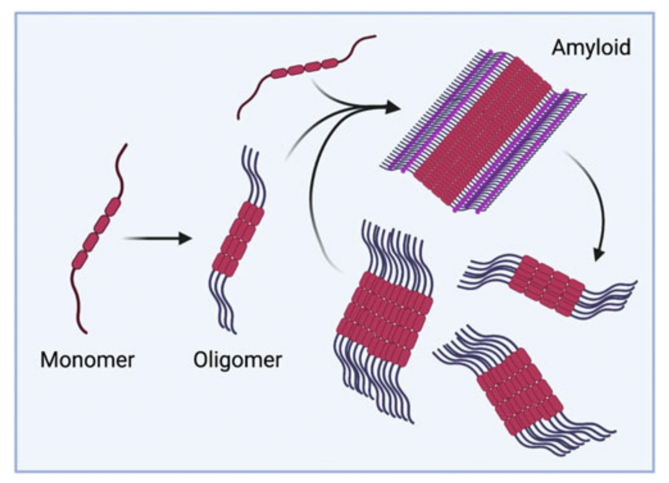

◀ **Abb. 1 :** Bildung anomaler Tau-Ablagerungen. Tau-Protein lagert sich zu Oligomeren und Amyloidfibrillen zusammen. Fibrillen binden lösliches Tau und induzieren wiederum dessen Fehlfaltung. Abbildung mit BioRender erstellt.

## Virale Proteine können die Übertragung von Proteinaggregaten begünstigen

Seit langem wird vermutet, dass virale Infektionen entzündliche oder toxische Reaktionen im Gehirn hervorrufen und somit die Entstehung oder den Verlauf neurodegenerativer Erkrankungen beeinflussen können [4]. Beispielhaft hierfür ist die spanische Grippe, bei der als Spätfolge einer Influenzainfektion parkinsonähnliche Symptome auftraten. Könnten Virusinfektionen auch die interzelluläre Ausbreitung von Proteinaggregaten beeinflussen? Viren sind intrazelluläre Erreger, die für ihre Verbreitung sowohl virale

Partikel freisetzen, aber auch EV und direkten Zellkontakt nutzen (**Abb. 2B**). Denkbar wäre etwa, dass virale Liganden, mit denen sich das Virus an die Zielzelle anheftet und mit dieser fusioniert, auch den Kontakt von Tau-Aggregat-Spenderzellen oder deren EV mit Empfängerzellen verstärken, wenn Viren die Spenderzelle befallen (**Abb. 2C**). So ist bekannt, dass die als Liganden dienenden Glykoproteine zahlreicher Viren auf der Zellmembran und EV infizierter Zellen vorliegen [5]. Um die interzelluläre Verbreitung fehlgefalteter Proteine und einen möglichen Einfluss viraler Glykoproteine zu untersuchen, wurde ein Zellkulturmodell basierend auf einem Spender-Empfänger-Prinzip verwendet. Hierzu wurden humane embryonale Nierenzellen (HEK) genetisch so modifiziert, dass sie die aggregationsanfälligen Domänen des Tau-Proteins, fusioniert mit grün fluoreszierendem Protein (GFP) oder FusionRed, bilden. Um entsprechende Spenderzellen zu generieren, wurden HEK-Tau-GFP-Zellen mit aggregiertem Tau aus ZNS-Proben von Tauopathiepatienten inkubiert [6]. Die durch die Zellen aufgenommenen Tau-Aggregate induzieren so die Bildung von Tau-GFP-Aggregaten (**Abb. 3**). Kommen Spender- und Empfängerzelle in direkten Kontakt, oder binden Spender-EV an die Empfängerzelle, bewirkt die Übertragung winziger Tau-GFP-Aggregate die Bildung von Tau-FusionRed-Aggregaten in den Empfängerzellen.

Um den Einfluss viraler Glykoproteine auf die Übertragung von Proteinaggregaten zu untersuchen, wurden Spenderzellen mit dem Glykoprotein VSV-G des vesikulären Stomatitisvirus oder Spike-S-Protein des SARS-CoV-2 ausgestattet. Der Rezeptor für VSV-G ist bereits auf HEK-Zellen vorhanden. Für die Anheftung des Spike-S an Empfängerzellen wurden diese gentechnisch so verändert, dass sie den Rezeptor ACE2 exprimieren. Die Ko-Kultivierung von Virusligand-tragenden Spenderzellen führte zu einem drastischen Anstieg von neu gebildeten Tau-Proteinaggregaten in Empfängerzellen. Die Induktion von Tau-Proteinaggregaten in Empfängern wurde auch stark erhöht durch die Inkubation von Empfängern mit EV, welche aus Spender-Zellkulturüberstand gewonnen wurden. Da VSV-G und Spike-S sowohl auf der Zellmembran des Spenders als auch auf dessen sezernierten EV nachweisbar war, lag nahe, dass die Glykoproteine, ähnlich wie bei Virusinfektionen, die Anheftung und Fusion zwischen Membranen des Spenders und der Zielzelle ermöglichten und so die Übertragung eines Tau-Aggregationskeims begünstigten. In der Tat verloren EV, deren Membran durch Ultraschallbehandlung zerstört wurde, ihre Wirksamkeit. Die Expression von VSV-G führte außerdem zu einer verstärkten Freisetzung von EV, die wiederum die Aggregatinduktion in der Empfängerzelle erhöhte.

Interessanterweise war der Effekt viraler Glykoproteine unabhängig vom Typ des Proteinaggregats. So spielte es keine Rolle, ob es sich um Tau-Aggregate handelte oder etwa um Prionen, infektiöse Proteinpartikel, die übertragbare spongiforme Enzephalopathien verursachen. Solange ein virales Glykoprotein auf Zelle oder EV vorhanden war, kam es zu einer effizienten Aggregatinduktion in der Empfängerzelle. Damit scheinen virale Glykoproteine, zumindest in Zellmodellen, die interzelluläre Übertragung krankheitsassoziierter Proteinpartikel zu erhöhen. Es bleibt zu zeigen, ob eine Infektion der Spenderzellen mit aktiven Viren ebenfalls Proteinaggregat-Übertragungen begünstigt. Dass dies der Fall sein könnte, zeigen Versuche mit Prionen, Proteinpartikeln bestehend aus fehlgefaltetem Prion Protein PrP. Hier führte eine zeitgleiche Infektion der Spenderzellen mit murinen Retroviren in Empfängern zu einer drastischen Zunahme der Prionlast [7].

## Erhöhte Viruslast bei Patienten mit Neurodegeneration?

Inwiefern akute oder chronische virale Infektionen des ZNS eine Rolle bei der progressiven Ausbreitung krankhafter Proteinaggregate spielen, muss weiter untersucht werden. Hier könnten auch das Alter des Patienten, in dem die Infektion erworben wird, wiederholte Infektionen oder reaktivierte latente Viren das Risiko der Ausbreitung von Proteinaggregaten beeinflussen. In der Tat scheinen Bestandteile von Herpes- oder Retroviren oder Antikörper, welche gegen sie gerichtet sind, in Patienten mit neurodegenerativen Veränderungen erhöht vorzuliegen [4]. Ebenfalls wird diskutiert, ob eine SARS-CoV-2-Infektion zu einer höheren Inzidenz neurodegenerativer Erkrankungen führen kann [8]. Weitere Studien sind nötig, um das komplexe Zusammenspiel viraler Infektio-

Hier steht
eine Anzeige.

Springer

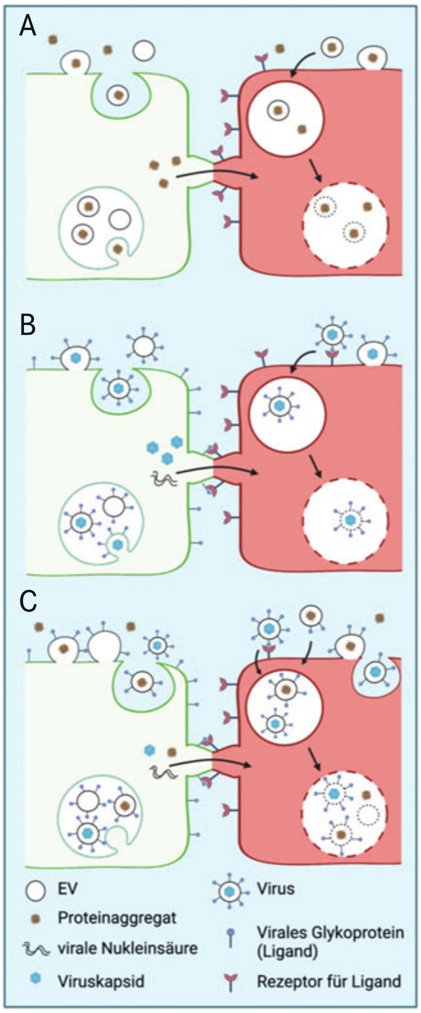

▲ **Abb. 2:** Einfluss viraler Proteine auf die interzelluläre Übertragung krankhafter Proteinaggregate. **A**, Übertragung von Proteinaggregaten zwischen Donor (grün) und Empfänger (rot) durch Freisetzung, direkten Zellkontakt oder EV. **B**, Freisetzung von membranumhüllten Viren und Virusbestandteilen. **C**, Virale Liganden auf Donorzelle und EV erhöhen die Übertragung krankheitsassoziierter Proteinaggregate zwischen Zellen. Abbildung mit BioRender erstellt.

▶ **Abb. 3:** Zellkulturmodelle zur Vermehrung von Tau-Proteinaggregaten. **A**, Herstellung von Spenderzellen mit grünen Tau-GFP-Aggregaten und Empfängerzellen mit löslichem Tau-FusionRed (rot). **B**, Spenderzellen oder deren EV werden mit Empfängerzellen kultiviert, die Tau-FusionRed produzieren. Die Übertragung winziger Tau-GFP-Aggregate führt zur Tau-FusionRed-Aggregation im Empfänger. Abbildung mit BioRender erstellt.

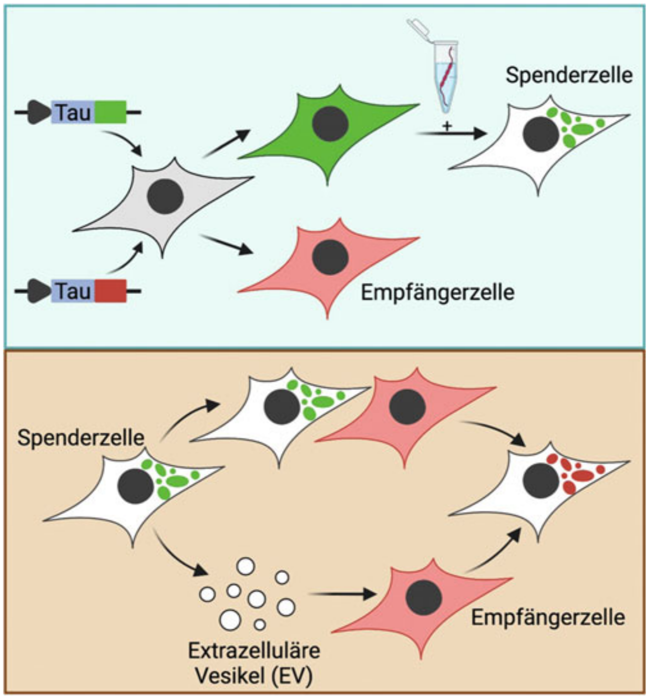

nen und neurodegenerativer Prozesse zu durchleuchten. Unsere Ergebnisse deuten jedoch darauf hin, dass virale Proteine bei der Übertragung von pathologischen Proteinaggregaten zwischen Zellen eine wichtige Rolle spielen könnten.


Funding note: Open Access funding enabled and organized by Projekt DEAL.

Stefanie-Elisabeth Heumüller 2004–2013 Biologiestudium an der Universität Göttingen. 2014–2019 Promotion am Zentrum für Biochemie der Universität Köln. Seit 2020 Postdoktorandin am Deutschen Zentrum für Neurodegenerative Erkrankungen (DZNE), Bonn.

Ina Maja Vorberg 1989–1995 Biologiestudium an der Universität Tübingen. 1996–1999 Promotion an der Bundesforschungsanstalt für Viruskrankheiten der Tiere, Tübingen. 1999–2003 Postdoktorandin an den Rocky Mountain Laboratories, NIH, Montana, USA. Von 2004–2010 Arbeitsgruppenleitung am Institut für Virologie, TU München. Seit 2010 und 2011 Arbeitsgruppenleitung am DZNE und Professorin an der Universität Bonn.

Danksagung

Wir danken unseren Kollegen für ihre Mitwirkung bei diesem Projekt. Lars Krüger, Marcus Neitzert und Dan Ehninger sind wir dankbar für hilfreiche Anmerkungen zu diesem Manuskript.


Literatur

[1] Thyrian JR Boekholt M Hoffmann W The prevalence of people with dementia in Germany-A nationwide analysis at the district level Nervenarzt 2020 91 1058 1061 10.1007/s00115-020-00923-y 32399609 PMC7606288
[2] Jucker M Walker LC Propagation and spread of pathogenic protein assemblies in neurodegenerative diseases Nat Neurosci 2018 21 1341 1349 10.1038/s41593-018-0238-6 30258241 PMC6375686
[3] Braak H Del Tredici K Alzheimer’s pathogenesis: is there neuron-to-neuron propagation? Acta Neuropathol 2011 121 589 595 10.1007/s00401-011-0825-z 21516512
[4] Zhou L Miranda-Saksena M Saksena NK Viruses and neurodegeneration Virol J 2013 10 172 10.1186/1743-422X-10-172 23724961 PMC3679988
[5] Yang L Li J Li S Extracellular vesicles regulated by viruses and antiviral strategies Front Cell Dev Biol 2021 9 722020 10.3389/fcell.2021.722020 34746122 PMC8566986
[6] Liu S Hossinger A Heumuller SE Highly efficient intercellular spreading of protein misfolding mediated by viral ligand-receptor interactions Nat Commun 2021 12 5739 10.1038/s41467-021-25855-2 34667166 PMC8526834
[7] Leblanc P Alais S Porto-Carreiro I Retrovirus infection strongly enhances scrapie infectivity release in cell culture EMBO J 2006 25 2674 2685 10.1038/sj.emboj.7601162 16724107 PMC1500854
[8] Krey L Huber MK Hoglinger GU Wegner F Can SARS-CoV-2 infection lead to neurodegeneration and Parkinson’s disease? Brain Sci 2021 11 1654 10.3390/brainsci11121654 34942956 PMC8699589
